# Relative Explanatory Contributions of Intrinsic Job Satisfaction, Affective Organizational Commitment, and Satisfaction with Financial Rewards to Turnover Intention Among Hospital Nurses: A Cross-Sectional Study

**DOI:** 10.3390/healthcare14182917

**Published:** 2026-09-09

**Authors:** Nozomu Takada, Seiko Tsuruta, Shoko Sugiyama, Kyoko Asakura

**Affiliations:** Graduate School of Medicine, Tohoku University, 2-1, Seiryo-machi, Aoba-ku, Sendai 980-8575, Miyagi, Japan; seiko.tsuruta.e8@tohoku.ac.jp (S.T.); s.sugiyama@tohoku.ac.jp (S.S.); asakura@tohoku.ac.jp (K.A.)

**Keywords:** turnover intention, nursing, intrinsic job satisfaction, organizational commitment, financial rewards, relative weight analysis

## Abstract

**Highlights:**

**What are the main findings?**

- Three job-related attitudes—intrinsic job satisfaction, affective organizational commitment, and satisfaction with financial rewards—were negatively associated with turnover intention among hospital nurses.
- Intrinsic job satisfaction and affective organizational commitment made the largest and comparable contributions to the explained variance in turnover intention, whereas satisfaction with financial rewards made a smaller but significant contribution.

**What are the implications of the main findings?**

- Intrinsic job satisfaction and affective organizational commitment showed the strongest associations with turnover intention among hospital nurses.
- Although its contribution was smaller than those of the psychological factors, satisfaction with financial rewards remained an independent contributor to turnover intention, suggesting that compensation should not be overlooked in retention strategies.

**Abstract:**

**Background:** Turnover among hospital nurses remains a major challenge for healthcare systems. Although various factors have been linked to turnover intention, few studies have examined them together. Therefore, we examined these factors within a single analytical model. **Methods:** In 2025, a cross-sectional survey was distributed through 70 hospitals randomly selected from the Tohoku and Kanto regions of Japan; individual nurses were selected at the discretion of each hospital, and data from 411 nurses were analysed. Turnover intention and affective organizational commitment were measured using previously validated scales, and intrinsic job satisfaction and satisfaction with financial rewards using scales developed for this study. The measurement model was examined using confirmatory factor analysis, which provided preliminary support for the two scales developed for this study. Multiple regression was performed, and relative weight analysis estimated each factor’s proportional contribution to the explained variance. **Results:** All three factors were significantly negatively associated with turnover intention. Relative weight analysis indicated that intrinsic job satisfaction accounted for the largest proportion of explained variance (44.49%), followed by affective organizational commitment (31.98%) and satisfaction with financial rewards (13.50%). **Conclusions:** Different job-related attitudes among nurses may contribute differently to turnover intention. The quality of work experiences and supportive organizational environments warrant evaluation as potential targets for future intervention research.

## 1. Introduction

Nurse turnover remains a persistent challenge for healthcare systems worldwide and has important implications for workforce sustainability and the quality of patient care. High turnover among hospital nurses increases recruitment and training costs, contributes to staff shortages, and disrupts the continuity of care. Research has also drawn attention to the “locked-in” state, in which employees remain in their current job despite low job satisfaction and a preference to leave because they perceive limited opportunities for alternative employment [1]; such situations are associated with negative consequences for employee well-being and health [2,3]. From an organizational perspective, understanding the factors associated with turnover intention is therefore essential for maintaining a sustainable and healthy workforce.

A substantial body of research has examined the determinants of turnover intention among nurses. Among the factors most frequently discussed are job satisfaction, organizational commitment, and compensation-related factors [4,5,6]. Job satisfaction reflects employees’ evaluation of their work experiences and has consistently been associated with turnover intention across occupational groups [4]. It is, however, a multifaceted construct, and its intrinsic facet—satisfaction derived from the content and experience of the work itself, rather than from the external conditions of employment—is especially relevant among nurses, whose work involves direct patient care and professional discretion [7,8]. The present study focused on this intrinsic facet. Affective organizational commitment represents the emotional attachment and identification that employees have with their organization [9]. Satisfaction with financial rewards reflects employee perceptions of the adequacy and fairness of compensation [10]. Each of these factors is associated with employee decisions to remain in or leave their positions.

We selected these three constructs on the basis of two theoretical perspectives. Herzberg’s two-factor theory distinguishes between intrinsic aspects of work, such as the content of the work itself and the sense of achievement it provides, and extrinsic conditions of employment, such as pay and job security; it proposes that these operate through different psychological mechanisms [7]. This distinction led us to include both intrinsic job satisfaction and satisfaction with financial rewards, which represent the intrinsic and economic domains of the employment experience, respectively. Social exchange theory, in turn, holds that employees who perceive their organization as valuing and supporting them reciprocate with emotional attachment and a sense of obligation to remain [11]. Affective organizational commitment was included to represent this relational dimension, which is directed toward the organization rather than the work or its material rewards. Together, these three constructs were intended to capture intrinsic, economic, and relational aspects of the employment experience within a single model.

Many other factors have been associated with nurses’ turnover intention: workload, staffing adequacy, burnout, leadership, work–family conflict, opportunities for professional development, shift patterns, psychological safety, and perceived organizational support. The present study did not attempt to model these factors comprehensively. Rather, it focused on three attitudinal constructs that are theoretically distinct, routinely assessed in organizational surveys, and potentially amenable to managerial action, examining their relative contributions when considered together.

Although each of these factors has been examined in relation to turnover intention, studies have often focused on a single domain or examined them separately [4,5,6]. Consequently, how these domains compare in their contribution to turnover intention, and particularly, whether satisfaction with financial rewards retains an independent contribution alongside psychological factors, remains unclear.

Therefore, in this study, we explored the associations of three job-related attitudes—intrinsic job satisfaction, affective organizational commitment, and satisfaction with financial rewards—with turnover intention among hospital nurses. Rather than determining causal priority among these constructs, we examined the relative explanatory contribution of each attitude to turnover intention within the same model. By considering these attitudes together, the study sought to provide exploratory insights into how different aspects of work experience may relate to intention to leave among nurses, along with how workplaces might support their continued employment and job satisfaction.

## 2. Materials and Methods

### 2.1. Study Design

This study employed a cross-sectional survey design and was conducted from August to September 2025.

### 2.2. Participants

The study population consisted of nursing professionals working in hospitals in Japan. Eligible participants were nurses holding a national licence as a registered nurse or a licensed practical nurse and currently employed at the participating hospitals, including those in supervisory positions. No minimum duration of employment was required. Nurses who were on leave of absence at the time of the survey were excluded.

The required sample size was estimated based on the total number of employed nurses in Japan (approximately 1.73 million) [12]. Using the sample size estimation method proposed by Krejcie and Morgan [13], the required sample size was calculated to be 384, assuming a confidence level of 95% and a margin of error of 5%. Because this approach is appropriate for estimating a population proportion rather than regression-based analyses, a sensitivity power analysis was additionally conducted using G*Power 3.1 [14]. With 411 participants, α = 0.05, and power of 0.80, the minimum detectable effect size was *f*^2^ = 0.04 (Δ*R*^2^ = 0.04) for the full model with nine predictors and *f*^2^ = 0.03 (Δ*R*^2^ = 0.03) for the three focal predictors. Both values correspond to small effects, indicating that the sample was large enough to detect effects of the magnitude typically observed in this research area.

Assuming a response rate of approximately 30%, the target sample size was set at 1300. The study sites were selected through random sampling from 577 hospitals located in the Tohoku and Kanto regions of Japan. A total of 70 hospitals were randomly selected. Depending on hospital size, 10–30 survey invitations were mailed to each hospital. In total, 1320 survey invitations were distributed. Invitation letters were sent to the nursing director of each participating hospital, who distributed them to nurses within the hospital. Each hospital selected individual nurses at its own discretion, and the research team specified no standardized within-hospital sampling procedure. Because the survey was anonymous and no hospital identifier was collected, we could not determine how many of the 70 hospitals yielded responses or how respondents were distributed across hospitals.

### 2.3. Measures

#### 2.3.1. Turnover Intention

Turnover intention was assessed using a six-item scale developed by Tei and Yamazaki [15], originally designed for workers in the information service industry. The scale measures multiple aspects of intention to leave the current workplace, including interest in leaving, consideration of job change, and potential job search behaviours. Each item was rated on a four-point Likert scale ranging from “strongly disagree” to “strongly agree,” with higher scores indicating stronger turnover intention.

This scale has been used in studies involving nursing professionals and is considered applicable to Japanese workers in general [16]. In this study, the Cronbach’s alpha coefficient for this scale was 0.91.

#### 2.3.2. Intrinsic Job Satisfaction

Intrinsic job satisfaction was measured using a three-item scale developed for this study based on prior research suggesting that certain characteristics of work influence intrinsic motivation and the perceived meaning of work [7,8]. The items were developed in Japanese for the present study and reviewed for content and clarity by the four co-authors, all of whom had expertise in nursing management or nursing research methodology. The scale was designed to capture satisfaction derived from the content and experience of the work itself rather than from the external conditions of employment. It included items assessing satisfaction with work content, sense of fulfilment, and the perceived value of continuing the current job. The responses were measured using a six-point Likert scale, with higher scores indicating greater intrinsic job satisfaction. The full wording of the items for both scales developed for the present study, together with English translations, is provided in Table S2. The Cronbach’s alpha coefficient for this scale in this study was 0.90.

#### 2.3.3. Affective Organizational Commitment

Affective organizational commitment was measured based on the three-component model of organizational commitment proposed by Allen and Meyer [9]. Affective commitment reflects the emotional attachment of employees to, and identification with, their organization. In this study, the Japanese version of the six-item scale developed by Takahashi [17] was used. Each item was rated on a six-point Likert scale ranging from “strongly disagree” to “strongly agree,” with higher scores indicating stronger affective commitment. The Cronbach’s alpha coefficient for this scale in this study was 0.90.

#### 2.3.4. Satisfaction with Financial Rewards

Satisfaction with financial rewards was measured using a scale developed for this study based on prior research regarding reward perception [18]. The items were developed in Japanese for the present study rather than translated from an existing non-Japanese measure. To develop the scale, an expert panel consisting of four researchers and practitioners with expertise in nursing management and labour management reviewed the items to ensure content validity and clarity. The scale consisted of five items assessing satisfaction with various aspects of financial compensation, including base salary, bonuses, allowances related to work duties (e.g., overtime pay, night shift allowances, and special duty allowances), salary increases, and overall earnings. The responses were measured using a six-point Likert scale ranging from “strongly disagree” to “strongly agree.” Higher scores indicated higher satisfaction with financial rewards. The Cronbach’s alpha coefficient for this scale in this study was 0.92.

#### 2.3.5. Demographic Variables

The measured demographic characteristics of the participants included age, sex, job position, marital status, presence of children, and annual income. Marital status was recorded in three categories (single, married, and other). Because the “other” category could not be interpreted unambiguously, these participants were combined with the “single” category for the regression analyses, yielding a binary variable (married versus single or other). Similarly, sex was recorded in three categories, and participants who selected “other” were combined with the female category (male versus female or other).

### 2.4. Statistical Analysis

For all four scales, total scores were analysed; no items were reverse-coded, and higher scores indicated higher levels of the respective constructs. Descriptive statistics were calculated for all study variables. Prior to the main analyses, the measurement model was examined using confirmatory factor analysis (CFA). A four-factor model was specified in which turnover intention, intrinsic job satisfaction, affective organizational commitment, and satisfaction with financial rewards were represented as correlated latent variables, with each item loading only on its intended factor. Because item skewness and kurtosis were within acceptable limits (|skewness| ≤ 0.48; |kurtosis| ≤ 1.15), the model was estimated using maximum likelihood. Model fit was evaluated using the comparative fit index (CFI), the Tucker–Lewis index (TLI), the root mean square error of approximation (RMSEA) with its 90% confidence interval, and the standardized root mean square residual (SRMR). Values of CFI and TLI ≥ 0.90, RMSEA ≤ 0.08, and SRMR ≤ 0.08 were taken to indicate acceptable fit [19]. The chi-square statistic was reported but not used as a primary criterion, because it is sensitive to sample size.

To evaluate discriminant validity and whether the observed associations reflected common measurement characteristics, the hypothesized four-factor model was compared with three alternative models: a single-factor model in which all items loaded on one general factor; a three-factor model in which intrinsic job satisfaction and turnover intention were combined into a single factor; and a three-factor model in which intrinsic job satisfaction and affective organizational commitment were combined. Because each alternative model was nested within the four-factor model, they were compared using chi-square difference tests and differences in CFI, with ΔCFI > 0.01 taken to indicate a substantive difference [20]. Composite reliability (CR) and average variance extracted (AVE) were calculated from the standardized factor loadings, and discriminant validity was further assessed using the Fornell–Larcker criterion, in which the square root of the AVE for each construct is compared with its correlations with the other constructs [21]. Modification indices were not used to re-specify the model, to preserve the confirmatory nature of the analysis. Harman’s single-factor test was additionally performed as a conventional check on common method bias [22].

Annual income was measured using a nine-category ordinal scale and was treated as a continuous variable in the main analysis. Because the categories were of approximately equal width, recoding them to category midpoints would be a linear transformation and yield identical standardized results.

Pearson’s correlation coefficients were computed to examine associations among intrinsic job satisfaction, affective organizational commitment, satisfaction with financial rewards, and turnover intention. Multiple regression analysis was conducted with turnover intention as the dependent variable and intrinsic job satisfaction, affective organizational commitment, and satisfaction with financial rewards as independent variables. Demographic variables were included as covariates because prior research suggests they may be associated with turnover intention. For the correlation and multiple regression analyses, statistical significance was set at *p* < 0.05. The assumptions of multiple regression were checked prior to interpretation. Linearity and homoscedasticity were assessed by inspecting plots of residuals against predicted values, and the normality of residuals was examined using a normal quantile plot; no substantive departures were observed. Influential observations were evaluated using Cook’s distance (maximum = 0.05), and multivariate outliers were assessed using Mahalanobis distance. No observations exceeded conventional thresholds for undue influence, and all 411 cases were retained in the analyses.

As the predictor variables were conceptually related and potentially correlated, relative importance analysis was conducted. This approach partitions the total explained variance (*R*^2^) of a regression model among predictors while accounting for correlations among them [23]. When predictors are correlated, standardized regression coefficients may not accurately reflect the relative contribution of each variable. Among available approaches, relative weight analysis (RWA) has been recommended as a useful method for estimating the relative contribution of correlated predictors to an outcome variable [24]. Accordingly, RWA was used in the present study.

Relative weights were calculated as the proportion of the total explained variance (*R*^2^) attributable to each predictor. Statistical analyses were conducted using JMP Student Edition 19.0 and the web-based RWA tool RWA-Web [25]. Bias-corrected and accelerated bootstrap 95% confidence intervals (CIs; corresponding to a two-tailed α of 0.05) for the raw relative weights were computed using 10,000 replications to indicate the precision of the estimates. The statistical significance of each relative weight was then evaluated using the procedure proposed by Tonidandel and LeBreton [24], in which each weight is compared with that of a randomly generated variable; a predictor was regarded as making a significant contribution to the explained variance when the 95% CI of this comparison did not include zero. Pairwise comparisons of relative weights were performed using the same bootstrap procedure. No adjustment for multiplicity was applied; therefore, these comparisons should be considered exploratory.

Two sensitivity analyses were conducted. First, one of the three intrinsic job satisfaction items referred to the perceived value of continuing in the current job, which could be regarded as conceptually close to turnover intention. This item was therefore removed, and intrinsic job satisfaction was recomputed from the remaining two items; internal consistency of the two-item measure was estimated with the Spearman–Brown coefficient, which is recommended over Cronbach’s alpha for two-item scales [26]. Second, to relax the assumption of equal intervals for annual income, the variable was dummy-coded into eight indicator variables, with the lowest category as the reference. All other aspects of the models, including covariates and estimation procedures, were identical to those of the main analysis.

### 2.5. Ethical Considerations

This study was conducted as an anonymous web-based survey, and careful consideration was given to protecting participant anonymity and privacy. Prior to participation, participants were informed of the purpose and procedures of the study, that participation was voluntary, that they could discontinue participation at any time without disadvantage, and that the collected data would be used solely for research purposes. Participants accessed the survey through a quick response code provided in printed materials containing the study information. After reviewing the study information on the web survey page, participants were able to proceed with the questionnaire only if they clicked the “I agree” button. This action was regarded as providing informed consent for participation in the study. The study protocol was reviewed and approved by the Ethics Committee of Tohoku University Graduate School of Medicine (approval number: 2025-1-361).

## 3. Results

### 3.1. Participant Characteristics

A total of 1320 survey invitations were distributed, and 412 responses were received (response rate: 31.2%). After excluding one incomplete response, data from 411 participants, which contained no item-level missing responses, were analysed (valid response rate: 31.1%). The demographic characteristics of the participants are presented in Table 1. The mean age of the participants was 42.6 years (standard deviation [SD] = 11.1), and 375 participants (91.2%) were female.

### 3.2. Measurement Model

The fit of the hypothesized four-factor model and the three alternative models is presented in Table 2. The four-factor model showed acceptable fit to the data (χ^2^(164) = 492.46, *p* < 0.001; CFI = 0.946; TLI = 0.937; RMSEA = 0.070, 90% CI [0.063, 0.077]; SRMR = 0.056). The chi-square statistic was significant, which is common with a sample of this size and a model of this complexity.

All three alternative models fitted the data substantially worse. The single-factor model showed poor fit (CFI = 0.555; TLI = 0.502; RMSEA = 0.196; SRMR = 0.133) and differed significantly from the four-factor model (Δχ^2^(6) = 2372.66, *p* < 0.001; ΔCFI = 0.391). The three-factor model combining intrinsic job satisfaction and turnover intention also fitted significantly worse (CFI = 0.852; RMSEA = 0.114; Δχ^2^(3) = 568.98, *p* < 0.001; ΔCFI = 0.094), as did the model combining intrinsic job satisfaction and affective organizational commitment (CFI = 0.864; RMSEA = 0.110; Δχ^2^(3) = 498.14, *p* < 0.001; ΔCFI = 0.082). These comparisons indicate that the four constructs were empirically distinguishable and, in particular, that intrinsic job satisfaction and turnover intention were not adequately represented by a single underlying factor.

Standardized factor loadings, composite reliability, and average variance extracted for the four-factor model are presented in Table 3. All factor loadings were significant (*p* < 0.001) and ranged from 0.60 to 0.93. Composite reliability ranged from 0.90 to 0.92 and average variance extracted from 0.61 to 0.76; both exceeded the conventional thresholds of 0.70 and 0.50, respectively. The three intrinsic job satisfaction items loaded at 0.77, 0.92, and 0.91 on their intended factor; the item concerning the perceived value of continuing in the current job loaded strongly on intrinsic job satisfaction rather than on turnover intention (Table S2).

For every pair of constructs, the square root of the average variance extracted exceeded the corresponding factor correlation, supporting discriminant validity (Table 3). The largest factor correlation was between intrinsic job satisfaction and affective organizational commitment (0.63), and the correlation between intrinsic job satisfaction and turnover intention was −0.59. Taken together, these findings provide preliminary support for the measurement properties of the two scales developed for this study. However, because the results were obtained from the same sample in which the scales were applied, full validation is not possible.

Harman’s single-factor test indicated that the first unrotated component accounted for 44.1% of the total variance. Because this test is a limited diagnostic, the poor fit of the single-factor confirmatory model provides stronger evidence against a single common factor accounting for the covariance structure, although it does not rule out common method variance more generally.

### 3.3. Associations Among Study Variables

To examine the bivariate relationships among the study variables, Pearson’s correlation coefficients were calculated. Subsequently, multiple regression analysis was conducted to assess the independent associations between each job-related factor and turnover intention while controlling for potential confounders.

The means, SDs, and correlation coefficients among the study variables are shown in Table 4. Intrinsic job satisfaction, affective organizational commitment, and satisfaction with financial rewards were all significantly negatively correlated with turnover intention (*r* = −0.56, −0.52, and −0.35, respectively; all *p* < 0.001).

### 3.4. Multiple Regression Analysis

Multiple regression analysis was conducted with turnover intention as the dependent variable. Intrinsic job satisfaction, affective organizational commitment, and satisfaction with financial rewards were entered as independent variables, whereas demographic characteristics were included as covariates. The results are presented in Table 5.

Intrinsic job satisfaction (β = −0.36, *p* < 0.001), affective organizational commitment (β = −0.23, *p* < 0.001), and satisfaction with financial rewards (β = −0.12, *p* = 0.01) were all significantly associated with turnover intention. None of the demographic variables showed a significant association with turnover intention. The adjusted coefficient of determination (adjusted *R*^2^) for the model was 0.38.

### 3.5. Relative Weight Analysis

RWA was conducted to estimate the proportional contribution of each predictor to the explained variance in turnover intention (*R*^2^ = 0.39; Table 5). Intrinsic job satisfaction accounted for the largest proportion, followed by affective organizational commitment and satisfaction with financial rewards. The bootstrap significance tests indicated that all three job-related factors contributed significantly to the explained variance, whereas none of the demographic covariates did.

Pairwise comparisons showed that the relative weights of intrinsic job satisfaction and affective organizational commitment did not differ significantly from each other (95% CI: [−0.118, 0.020]). However, both factors had significantly larger relative weights than did satisfaction with financial rewards (intrinsic job satisfaction vs. financial rewards: 95% CI: [0.057, 0.190]; affective organizational commitment vs. financial rewards: 95% CI: [0.020, 0.128]). Notably, although satisfaction with financial rewards showed the smallest relative weight among the three job-related factors, it remained significant and exceeded the combined contribution of all demographic variables.

### 3.6. Sensitivity Analysis

At the item level, the intrinsic job satisfaction item concerning the perceived value of continuing in the current job correlated with turnover intention at a magnitude comparable to that of the other two items (*r* = −0.53, versus −0.52 for satisfaction with work content and −0.47 for sense of fulfilment; all *p* < 0.001), and it correlated closely with the full three-item composite (*r* = 0.93). The two-item composite showed adequate internal consistency (Spearman–Brown coefficient = 0.83) and correlated strongly with the original three-item composite (*r* = 0.98).

When the two-item measure was substituted for the original, the regression model explained a comparable proportion of variance (*R*^2^ = 0.38, adjusted *R*^2^ = 0.37), and all three job-related factors remained significantly associated with turnover intention (intrinsic job satisfaction, β = −0.33, *p* < 0.001; affective organizational commitment, β = −0.26, *p* < 0.001; satisfaction with financial rewards, β = −0.11, *p* = 0.01). In the relative weight analysis, intrinsic job satisfaction accounted for 41.56% of the explained variance (44.49% in the main analysis), affective organizational commitment for 34.00% (31.98%), and satisfaction with financial rewards for 13.84% (13.50%). All three weights remained significant, and the pattern of pairwise differences was unchanged: the relative weights of intrinsic job satisfaction and affective organizational commitment did not differ significantly from each other (95% CI: [−0.097, 0.041]), whereas both were significantly larger than that of satisfaction with financial rewards (95% CI: [0.039, 0.172] and [0.025, 0.130], respectively). Detailed results are presented in Table S1.

The three-item measure was retained for the primary analysis on both empirical and conceptual grounds. In the confirmatory factor analysis, the item concerning the perceived value of continuing in the current job had a standardized loading of 0.91 on intrinsic job satisfaction, a value comparable to the item on sense of fulfilment (0.92) and higher than that of the item on satisfaction with work content (0.77). Thus, this item functioned as a core rather than a peripheral indicator of the construct. Its correlation with turnover intention (*r* = −0.53) was comparable to that of the item on satisfaction with work content (*r* = −0.52) and did not stand out from *r* values of the other two items. Retaining all three items also preserves the content breadth of the construct as originally specified, given that this item captures an evaluative aspect of intrinsic satisfaction that the remaining two items do not.

A second sensitivity analysis examined the treatment of annual income. When income was dummy-coded into eight indicator variables rather than treated as continuous, the standardized coefficients for the three focal predictors were essentially unchanged (intrinsic job satisfaction, β = −0.36; affective organizational commitment, β = −0.23; satisfaction with financial rewards, β = −0.11; all *p* ≤ 0.01), and the overall effect of income remained non-significant (*F*(8, 394) = 1.29, *p* = 0.25). Although *R*^2^ increased from 0.39 to 0.40 owing to the additional degrees of freedom, the adjusted *R*^2^ was virtually identical (0.380 versus 0.379). In the relative weight analysis, the raw relative weights of the three focal predictors were also essentially unchanged (intrinsic job satisfaction, 0.174 versus 0.175; affective organizational commitment, 0.124 versus 0.125; satisfaction with financial rewards, 0.052 versus 0.053), and their rank order was identical to that in the main analysis.

## 4. Discussion

In this study, we examined the associations between three job-related attitudes—intrinsic job satisfaction, affective organizational commitment, and satisfaction with financial rewards—and turnover intention among hospital nurses. All three factors were negatively associated with turnover intention. Relative weight analysis further indicated that intrinsic job satisfaction and affective organizational commitment made the largest and comparable contributions to the explained variance in turnover intention, whereas satisfaction with financial rewards made a smaller but significant contribution.

These findings suggest that different domains of work experience play distinct roles in shaping nurses’ intentions to leave their jobs. Although satisfaction with financial rewards showed a significant association with turnover intention, intrinsic job satisfaction and affective organizational commitment accounted for larger proportions of the explained variance. This pattern indicates that, when these factors are examined within the same model, evaluations of the work itself and emotional attachment to the organization relate more closely to turnover intention than do perceptions of financial compensation. This interpretation should be treated with caution, given that within-hospital clustering could not be examined and the standard errors reported here may therefore be underestimated—a consideration most relevant to satisfaction with financial rewards, for which the observed association was comparatively small.

Studies have consistently reported that job satisfaction and organizational commitment are important correlates of turnover intention among nurses and other healthcare professionals [4,5,6]. The present study focused specifically on the intrinsic facet of job satisfaction, and the observed pattern is consistent with this broader literature. For example, research has shown that positive work experiences and supportive work environments are associated with lower intentions to leave among healthcare workers [5]. Similarly, affective organizational commitment is often linked to stronger intentions to remain in the organization [9]. The associations reported here are therefore consistent with a substantial body of existing evidence. The incremental contribution of the present study lies in examining these three attitudes simultaneously within a single model and estimating their relative explanatory contributions, rather than in the direction of the associations themselves.

It should be noted, however, that the results of this study do not imply that financial compensation is unimportant. Satisfaction with financial rewards remained significantly associated with turnover intention even after controlling for the other variables. Moreover, in the RWA, its contribution was significant and numerically exceeded the combined contribution of all demographic variables, indicating that satisfaction with financial rewards is a meaningful and independent correlate of turnover intention, rather than a negligible one. Previous research has also reported that compensation-related perceptions are associated with employee turnover intentions [4]. Although compensation has been discussed as one component of a broader set of work-related resources [10], the present study did not examine how financial rewards operate in combination with other aspects of the work environment, which remains an important question for future research.

From a practical perspective, the present findings may inform how workplaces consider turnover intention among nurses, although several caveats apply. Relative importance in a cross-sectional regression model reflects the proportion of explained variance attributable to each predictor; it does not establish causal priority, nor does it indicate which factors are most modifiable, most responsive to intervention, or most cost-effective to address. Accordingly, the finding that intrinsic job satisfaction and affective organizational commitment accounted for larger proportions of the explained variance should not be read as evidence that interventions targeting these attitudes would produce correspondingly larger reductions in turnover intention. Rather, the present results are best regarded as hypothesis-generating for future longitudinal and intervention research—for example, that enhancing meaningful engagement with work tasks and strengthening supportive organizational environments may be worth evaluating [27,28].

The locked-in concept was introduced above only as contextual background and was not examined empirically in the present study, which did not measure perceived employment alternatives. Whether the attitudes examined here are also relevant to locked-in situations remains a question for future research.

At the same time, financial compensation should not be overlooked in turnover prevention strategies. Although its relative explanatory contribution was smaller than that of the other factors in this study, compensation remains an important component of employment conditions. Previous research has shown that perceptions of distributive justice and reward imbalance are associated with turnover intention among healthcare workers [29,30]. In contexts such as Japan, where seniority-based wage systems are common [31], the perceived adequacy of compensation may be shaped by structural features of the wage system. Accordingly, efforts to address turnover intention should consider not only pay levels but also the transparency and perceived fairness of compensation systems.

This study has some limitations. First, the cross-sectional design and reliance on self-reported data limit the ability to draw causal conclusions and may introduce common method bias. Future longitudinal or multi-source studies would help clarify the relationships between work-related factors and turnover intention. Although both Harman’s single-factor test and the poor fit of the single-factor confirmatory model indicated that common method variance was unlikely to account for the findings, neither approach fully rules out its influence, and more rigorous designs (e.g., marker variable or common latent factor approaches) should be considered in future studies. Second, although the response rate (31.2%) was within the typical range for surveys of nurses in Japan, the possibility of nonresponse bias cannot be excluded, because the characteristics of nonrespondents could not be assessed. The proportion of female participants (91.2%) was comparable to that among employed nurses nationally (92.4%) [12], although the national statistics include nurses working outside hospitals and are not disaggregated by region. Third, although the hospitals were randomly selected, the nurses within each hospital were not, and the absence of hospital identifiers meant that clustering of responses within hospitals could not be examined. Because nurses in the same hospital share organizational conditions, their responses may not have been fully independent, and the standard errors reported here may be underestimated.

Fourth, the relative weight analysis estimates the proportional contribution of predictors to the explained variance of a regression model but does not indicate causal priority or temporal ordering among variables. The relative weights reported here are conditional on the predictors included in the model and would change if other established correlates of turnover intention were included; they should therefore not be interpreted as absolute estimates of the importance of these constructs. In particular, several occupational factors that may be associated with turnover intention—including clinical experience, tenure, department, shift pattern, employment type, hospital size, and workload—were not measured; therefore, residual confounding by these factors cannot be excluded. Fifth, locked-in status was not directly assessed in this study; references to the locked-in phenomenon therefore reflect a conceptual interpretation rather than an empirical assessment.

Sixth, two of the scales used in this study (intrinsic job satisfaction and satisfaction with financial rewards) were developed for the present research. Although the confirmatory factor analysis supported the hypothesized four-factor structure, and both scales demonstrated adequate composite reliability, convergent validity, and discriminant validity, these analyses were conducted within the same sample in which the scales were applied. Independent replication and examination of criterion-related validity against established external measures would further strengthen confidence in these instruments. In addition, the four-factor model showed acceptable rather than excellent fit, and the model was deliberately not re-specified using modification indices to preserve the confirmatory nature of the analysis. The scales were not pilot-tested before administration, and their content validity was established solely through review by the research team. Seventh, because the intrinsic job satisfaction measure captured only the intrinsic facet of job satisfaction, other facets such as satisfaction with supervision, co-workers, or promotion opportunities were not assessed; the relative weight reported here should therefore be understood as pertaining specifically to the intrinsic domain. Finally, the participants were limited to hospital nurses in Japan, and therefore caution is needed when generalizing the findings to other healthcare settings or countries.

## 5. Conclusions

This study examined the associations of three job-related attitudes—intrinsic job satisfaction, affective organizational commitment, and satisfaction with financial rewards—with turnover intention among hospital nurses in Japan. All three factors were negatively associated with turnover intention. Relative weight analysis further indicated that intrinsic job satisfaction and affective organizational commitment made the largest and comparable contributions to the explained variance, whereas satisfaction with financial rewards made a smaller but significant and independent contribution. These findings suggest that different aspects of work experiences among nurses may contribute differently to turnover intention when examined within the same analytical framework. These patterns indicate that improving the quality of nurses’ work experiences and strengthening supportive organizational environments merit evaluation as potential approaches to reducing turnover intention. Future research using longitudinal designs would help clarify how changes in workplace factors influence turnover intention over time.

## Figures and Tables

**Table 1:** Participant characteristics (*n* = 411).

Variable	Category	*n* (%) or Mean (SD)
Age (years)		42.6 (11.1)
	Range	21–76
Sex	Male	35 (8.5)
	Female	375 (91.2)
	Other	1 (0.2)
Position	Staff nurse	293 (71.3)
	Assistant head nurse/head nurse	118 (28.7)
Marital status	Single	137 (33.3)
	Married	254 (61.8)
	Other	20 (4.9)
Children	No children	175 (42.6)
	Has children	236 (57.4)
Annual income (JPY)	<3 million	44 (10.7)
	3–4 million	87 (21.1)
	4–5 million	104 (25.3)
	5–6 million	96 (23.4)
	6–7 million	55 (13.4)
	7–8 million	15 (3.7)
	8–9 million	8 (1.9)
	9–10 million	1 (0.2)
	≥10 million	1 (0.2)

Note: Values are *n* (%) for categorical variables and mean (SD) for age. Percentages may not sum to 100% because of rounding. SD, standard deviation.

**Table 2:** Fit indices for the hypothesized measurement model and alternative models (*n* = 411).

Model	χ^2^	*df*	CFI	TLI	RMSEA [90% CI]	SRMR	Δχ^2^	Δ*df*	ΔCFI
Four-factor model (hypothesized)	492.46	164	0.946	0.937	0.070 [0.063, 0.077]	0.056	—	—	—
Single-factor model	2865.12	170	0.555	0.502	0.196 [0.190, 0.203]	0.133	2372.66 ***	6	0.391
Three-factor model (IJS and TI combined)	1061.44	167	0.852	0.832	0.114 [0.108, 0.121]	0.082	568.98 ***	3	0.094
Three-factor model (IJS and AOC combined)	990.60	167	0.864	0.845	0.110 [0.103, 0.116]	0.068	498.14 ***	3	0.082

Note: IJS, intrinsic job satisfaction; TI, turnover intention; AOC, affective organizational commitment; CFI, comparative fit index; TLI, Tucker–Lewis index; RMSEA, root mean square error of approximation; CI, confidence interval; SRMR, standardized root mean square residual. Δχ^2^, Δ*df*, and ΔCFI refer to the comparison with the hypothesized four-factor model, within which each alternative model is nested. All models were estimated using maximum likelihood. The chi-square statistic was significant for all models (*p* < 0.001). *** *p* < 0.001.

**Table 3:** Standardized factor loadings, composite reliability, average variance extracted, and factor correlations for the four-factor measurement model (*n* = 411).

**Panel A.** Factor loadings and construct reliability				
**Construct**	**No. of items**	**Standardized loadings**	**CR**	**AVE**
Turnover intention	6	0.69–0.85	0.912	0.633
Intrinsic job satisfaction	3	0.77–0.92	0.903	0.758
Affective organizational commitment	6	0.60–0.90	0.901	0.607
Satisfaction with financial rewards	5	0.74–0.93	0.921	0.703
**Panel B.** Factor correlations and discriminant validity				
	**1**	**2**	**3**	**4**
1. Turnover intention	**0.796**			
2. Intrinsic job satisfaction	−0.585	**0.871**		
3. Affective organizational commitment	−0.588	0.628	**0.779**	
4. Satisfaction with financial rewards	−0.383	0.390	0.460	**0.838**

Note: CR, composite reliability; AVE, average variance extracted. All factor loadings were significant at *p* < 0.001. In Panel B, values on the diagonal (in bold) are the square roots of the AVE; off-diagonal values are correlations between latent factors. Discriminant validity is supported when the diagonal value for each construct exceeds its correlations with the other constructs.

**Table 4:** Means, SDs, and correlations among study variables.

	Mean	SD	1	2	3	4
1. Turnover intention	16.0	5.0	-			
2. Intrinsic job satisfaction	10.9	3.2	−0.56 ***	-		
3. Affective organizational commitment	17.5	6.0	−0.52 ***	0.60 ***	-	
4. Satisfaction with financial rewards	12.3	5.4	−0.35 ***	0.39 ***	0.42 ***	-

Note: Values below the diagonal represent Pearson’s correlation coefficients. The theoretical score ranges were 6–24 for turnover intention, 3–18 for intrinsic job satisfaction, 6–36 for affective organizational commitment, and 5–30 for satisfaction with financial rewards. *** *p* < 0.001. SD, standard deviation.

**Table 5:** Multiple regression results and relative weights (RWA) for turnover intention.

Variable	B [95% CI]	SE	β	*t*	*p*-Value	VIF	Raw Relative Weight [95% CI]	Relative Weight (%)
Age	−0.04 [−0.09, 0.00]	0.02	−0.09	−1.88	0.06	1.67	0.014 [0.003, 0.036]	3.54
Sex (male)	−0.49 [−1.19, 0.21]	0.36	−0.06	−1.39	0.17	1.04	0.002 [0.000, 0.009]	0.44
Position (assistant head nurse/head nurse)	0.41 [−0.11, 0.92]	0.26	0.07	1.56	0.11	1.48	0.003 [0.001, 0.004]	0.72
Marital status (married)	0.15 [−0.36, 0.66]	0.26	0.03	0.59	0.56	1.67	0.001 [0.000, 0.003]	0.37
Presence of children (has children)	−0.28 [−0.84, 0.28]	0.29	−0.06	−0.97	0.33	2.10	0.007 [0.001, 0.020]	1.75
Annual income	−0.25 [−0.55, 0.06]	0.15	−0.07	−1.60	0.11	1.34	0.013 [0.002, 0.034]	3.19
Intrinsic job satisfaction	−0.55 [−0.71, −0.40]	0.08	−0.36	−7.09	<0.001 ***	1.70	0.175 [0.119, 0.232] ^†^	44.49
Affective organizational commitment	−0.20 [−0.28, −0.11]	0.04	−0.23	−4.42	<0.001 ***	1.85	0.125 [0.083, 0.172] ^†^	31.98
Satisfaction with financial rewards	−0.11 [−0.19, −0.03]	0.04	−0.12	−2.60	0.01 *	1.31	0.053 [0.026, 0.086] ^†^	13.50

Note: B, unstandardized regression coefficient; SE, standard error; β, standardized regression coefficient; *t*, *t* value; VIF, variance inflation factor; CI, confidence interval. Reference categories: sex, female or other; position, staff nurse; marital status, single or other; and presence of children, no children. Asterisks denote the statistical significance of the regression coefficients: * *p* < 0.05, *** *p* < 0.001. Relative weights represent the proportion of the total explained variance (*R*^2^) attributable to each predictor. Confidence intervals for the raw relative weights were bias-corrected and accelerated bootstrap intervals based on 10,000 replications. ^†^ denotes a relative weight that was significant, as determined by comparison with a randomly generated variable (the 95% CI of the comparison did not include zero). The overall model was significant, *F*(9, 401) = 28.74, *p* < 0.001; *R*^2^ = 0.39, adjusted *R*^2^ = 0.38; *n* = 411.

## Data Availability

The data presented in this study are available on request from the corresponding author. The data are not publicly available due to privacy considerations and the terms of informed consent, under which participants agreed to the use of their data solely for the purposes of this research.

## Supplementary Materials

The following supporting information can be downloaded at https://www.mdpi.com/article/10.3390/healthcare14182917/s1, Table S1: Multiple regression results and relative weights for turnover intention: main analysis (three-item intrinsic job satisfaction measure) versus sensitivity analysis (two-item measure), with pairwise comparisons of relative weights; Table S2: Items of the scales developed for the present study, with standardized factor loadings from the confirmatory factor analysis (*n* = 411).

## Abbreviations

The following abbreviations are used in this manuscript:

AOC	Affective organizational commitment
AVE	Average variance extracted
CFA	Confirmatory factor analysis
CFI	Comparative fit index
CI	Confidence interval
CR	Composite reliability
IJS	Intrinsic job satisfaction
RMSEA	Root mean square error of approximation
RWA	Relative weight analysis
SD	Standard deviation
SRMR	Standardized root mean square residual
TI	Turnover intention
TLI	Tucker–Lewis index
VIF	Variance inflation factor
