# Supplementary material for: Relative Explanatory Contributions of Intrinsic Job Satisfaction, Affective Organizational Commitment, and Satisfaction with Financial Rewards to Turnover Intention Among Hospital Nurses: A Cross-Sectional Study
Source: Healthcare (Basel). 2026 Sep 9;14(18):2917. doi: 10.3390/healthcare14182917 (PMC13606346; doi:10.3390/healthcare14182917)
Supplement: Supplementary file 1 [file healthcare-14-02917-s001.zip › healthcare-4418415-supplementary.pdf]

1. Supplementary Table S1

Table S1. Multiple regression results and relative weights for turnover intention: main analysis (three-item intrinsic job satisfaction measure) versus sensitivity analysis (two-item measure).

Variable	Main: β	Main: raw RW [95% CI]	Main: %R^2	Sens: β	Sens: raw RW [95% CI]	Sens: %R²
Age	−0.09	0.014 [0.003, 0.036]	3.54	−0.09	0.014 [0.002, 0.035]	3.55
Sex (male)	−0.06	0.002 [0.000, 0.009]	0.44	−0.05	0.002 [0.000, 0.009]	0.38
Position (assistant head nurse/head nurse)	0.07	0.003 [0.001, 0.004]	0.72	0.08	0.003 [0.001, 0.004]	0.77
Marital status (married)	0.03	0.001 [0.000, 0.003]	0.37	0.04	0.002 [0.000, 0.003]	0.39
Presence of children (has children)	−0.06	0.007 [0.001, 0.020]	1.75	−0.07	0.007 [0.001, 0.021]	1.93
Annual income	−0.07	0.013 [0.002, 0.034]	3.19	−0.08	0.014 [0.002, 0.037]	3.57
Intrinsic job satisfaction	−0.36 ***	0.175 [0.119, 0.232] †	44.49	−0.33 ***	0.158 [0.103, 0.217] †	41.56
Affective organizational commitment	−0.23 ***	0.125 [0.083, 0.172] †	31.98	−0.26 ***	0.129 [0.088, 0.177] †	34.00
Satisfaction with financial rewards	−0.12 *	0.053 [0.026, 0.086] †	13.50	−0.11 *	0.053 [0.026, 0.086] †	13.84
Total R^2		0.39			0.38	
Adjusted R^2		0.38			0.37	

Note: RW, relative weight; CI, confidence interval. The main analysis used the original three-item intrinsic job satisfaction measure; the sensitivity analysis used a two-item measure, dropping the item on the perceived value of continuing in the current job. All other model specifications were identical. Reference categories: sex, female or other; position, staff nurse; marital status, single or other; and presence of children, no children. * $p < 0.05$, *** $p < 0.001$ for regression coefficients. † denotes a relative weight that was significant, as determined by comparison with a randomly generated variable (the 95% CI of the comparison did not include zero). The 95% CIs for raw relative weights are bias-corrected and accelerated bootstrap intervals based on 10,000 replications.

Table S1 (continued). Pairwise comparisons of relative weights

Comparison	Main analysis 95% CI	Sensitivity analysis 95% CI	Conclusion
Intrinsic job satisfaction vs. affective organizational commitment	[−0.118, 0.020]	[−0.097, 0.041]	Not significantly different (both)
Intrinsic job satisfaction vs. satisfaction with financial rewards	[0.057, 0.190]	[0.039, 0.172]	Significantly different (both)
Affective organizational commitment vs. satisfaction with financial rewards	[0.020, 0.128]	[0.025, 0.130]	Significantly different (both)

2. Supplementary Table S2

Table S2. Items of the scales developed for the present study, with standardized factor loadings from the confirmatory factor analysis ($n = 411$).

Construct	Item	Japanese original	English translation	Standardized loading
Intrinsic job satisfaction	1	私は現在の仕事の内容に満足している	I am satisfied with the content of my current work.	0.774
	2	私は自分の仕事にやりがいを感じている	I find my work rewarding.	0.919
	3	この仕事は、私にとって続ける価値があると思う	I believe this work is worth continuing for me.	0.911
Satisfaction with financial rewards	1	私は現在の基本給に満足している	I am satisfied with my current base salary.	0.898
	2	私は現在の賞与に満足している	I am satisfied with my current bonuses.	0.842
	3	私は現在の業務内容に応じた手当（超過勤務手当、夜勤手当、特殊勤務手当など）に満足している	I am satisfied with the allowances I receive for my work duties (e.g., overtime, night-shift, and special-duty allowances).	0.735
	4	私は最近の昇給に満足している	I am satisfied with my recent pay raises.	0.770
	5	私は現在の給与の総支給額に満足している	I am satisfied with my current total gross pay.	0.930

Note: Both scales were developed for the present study and were administered in Japanese; the English translations are provided for reference only and were not used in data collection. All items were rated on a six-point Likert scale, with higher scores indicating greater satisfaction. Standardized factor loadings are taken from the four-factor confirmatory factor analysis reported in Table 3; all loadings were significant at $p < 0.001$. Items for turnover intention and affective organizational commitment were drawn from previously published scales and are not reproduced here; see the original sources [15] and [17].
